# The effect of light quality on plant physiology, photosynthetic, and stress response in *Arabidopsis thaliana* leaves

**DOI:** 10.1371/journal.pone.0247380

**Published:** 2021-03-04

**Authors:** Nafiseh Yavari, Rajiv Tripathi, Bo-Sen Wu, Sarah MacPherson, Jaswinder Singh, Mark Lefsrud

**Affiliations:** 1 Department of Bioresource Engineering, McGill University–Macdonald Campus, Sainte-Anne-de-Bellevue, Quebec, Canada; 2 Department of Plant Science, McGill University–Macdonald Campus, Sainte-Anne-de-Bellevue, Quebec, Canada; National Taiwan University, TAIWAN

## Abstract

The impacts of wavelengths in 500–600 nm on plant response and their underlying mechanisms remain elusive and required further investigation. Here, we investigated the effect of light quality on leaf area growth, biomass, pigments content, and net photosynthetic rate (Pn) across three *Arabidopsis thaliana* accessions, along with changes in transcription, photosynthates content, and antioxidative enzyme activity. Eleven-leaves plants were treated with BL; 450 nm, AL; 595 nm, RL; 650 nm, and FL; 400–700 nm as control. RL significantly increased leaf area growth, biomass, and promoted Pn. BL increased leaf area growth, carotenoid and anthocyanin content. AL significantly reduced leaf area growth and biomass, while Pn remained unaffected. Petiole elongation was further observed across accessions under AL. To explore the underlying mechanisms under AL, expression of key marker genes involved in light-responsive photosynthetic reaction, enzymatic activity of antioxidants, and content of photosynthates were monitored in Col-0 under AL, RL (as contrast), and FL (as control). AL induced transcription of *GSH2* and *PSBA*, while downregulated *NPQ1* and *FNR2*. Photosynthates, including proteins and starches, showed lower content under AL. SOD and APX showed enhanced enzymatic activity under AL. These results provide insight into physiological and photosynthetic responses to light quality, in addition to identifying putative protective-mechanisms that may be induced to cope with lighting-stress in order to enhance plant stress tolerance.

## Introduction

Among various environmental factors, light is one of the most important variables affecting photosynthesis as well as plant growth and development [1]. Plants require light not only as an energy source but also as a clue to adjust their development to environmental conditions. During photosynthesis, absorbed energy is transferred to the photosynthetic apparatus, which is comprised of Photosystem I (PSI), Photosystem II (PSII), electron transport ‬carriers (cytochrome b6f (cytb6f), plastoquinone (PQ), plastocyanin (PC)), and ATP synthase. The light-responsive photosynthetic process is driven by the released electrons through the water-splitting reaction on the PSII side, followed by NADP^+^ reduction to NADPH, and proton flow into the lumen in order to generate ATP. Generated NADPH and ATP serve as an energy source for the carbon fixation process [2].‬‬‬‬‬‬‬‬‬‬‬‬‬‬‬‬‬‬‬‬‬‬‬‬‬‬‬‬‬‬‬‬‬‬‬‬‬‬‬‬‬‬‬‬‬‬‬‬‬‬‬‬‬‬‬‬‬‬‬‬‬‬‬‬‬‬‬‬‬‬‬‬‬‬‬‬‬‬‬‬‬‬‬‬‬‬‬‬‬

‬‬‬‬‬‬‬‬‬‬‬‬ Both quality and quantity of incident light can have drastic impacts on photosynthetic activity and photosystem adaption to changing light quality [3, 4]. Earlier studies on photosynthetic activity reported that photosynthesis is a wavelength-dependent response, in which amber light (AL; 595 nm) induces higher photosynthetic rates than blue light (BL; 450 nm) or red light (RL; 650 nm) [3, 5, 6]. These studies have become the foundation for our plant lighting research as light emitting diodes (LEDs) are proven to be ‬an ‬optimal and effective ‬tool to study the effect of ‬‬‬‬wavelength on plant physiological and biochemical responses [7–10]. Prior research has demonstrated that the wavelength range from 430–500 nm is effective at simulating pigmentation, metabolism of secondary metabolites, photosynthetic function, and development of chloroplasts [11–14]. The wavelength range of 640–670 nm was found effective in promoting photosynthetic activity, plant biomass and leaf area growth [3, 15] while playing critically important roles in the development of photosynthetic apparatus, net photosynthetic rate (Pn) and primary metabolism [12, 16]. Growing research on the wavelength range 500–600 nm have highlighted its important physiological and morphological impact on growth, chlorophyll content, and photosynthetic function [8, 17–19]. However, conflicting results on the impact of AL were reported [3, 20]. Although AL results in high photosynthetic activity, poor plant growth responses such as elongation and growth suppression have been reported [20, 21], and this underlying mechanism remains unknown. In addition to this, AL is weakly absorbed by the photosynthetic pigments [22]. At the current state, further investigation of AL impact is required to better understand the photoactivity of the photosystems.‬ ‬‬‬‬‬‬‬‬‬‬‬‬‬‬‬‬‬‬‬‬‬‬‬‬‬‬‬‬‬‬‬‬‬‬‬‬‬‬‬‬‬‬‬‬‬‬‬‬‬‬‬‬‬‬‬‬‬

Recent studies reported that light quality and quantity can have drastic impacts on imbalanced excitation of either PSII or PSI, resulting in energy imbalance between photosystems and triggering stoichiometric adjustments of photosynthetic complexes [23, 24]. This imbalance between the two photosystems can result in generation of harmful reactive intermediates, mainly reactive oxygen species (ROS) [25, 26]. Generation of ROS can result in oxidative damage to the chloroplasts, leading to photosystem photo-inhibition that strongly limits plant growth [27].‬‬‬‬ To maintain steady state photosynthetic efficiency and prevent ROS accumulation, plants activate the buffering mechanisms, including cyclic photosynthetic electron flow (CEF) and non-photochemical quenching (NPQ) [28, 29]. To scavenge ROS, plants further stimulate antioxidative mechanisms via enhanced activity of associated enzymes such as glutathione synthetase (GSH), ascorbate peroxidase (APX), and superoxide dismutase (SOD) [30]. These studies and their findings allow us to understand the impact of light within photosystems; however, the wavelength that can induce such stress responses and their physiological consequence on plants remain poorly studied.

Therefore, to better understand the effect of light quality on plant growth and photosynthetic performance, we studied three narrow-wavelength LEDs of blue light (BL; 450 nm), amber light (AL; 595 nm), and red light (RL; 650 nm), and compared them with fluorescent light (FL; 400–700) as the control. We chose light quality of BL and RL as leaf pigments have absorption peaks at these wavelengths [31]. AL was chosen due to the conflicting results between high photosynthetic activity and poor plant growth responses [3, 5]. Furthermore, to assess whether light quality-induced changes in plant growth and photosynthesis are mediated by the genotype, we investigated the light quality response in three *A*. *thaliana* accessions Col-0, Est-1, and C24. These accessions show different geographical distributions and hence are adopted to different environments [32–34]. Congruently, they show a high degree of divergence in their photosynthetic response to the light environment [35, 36]‬‬‬‬‬‬‬‬‬‬‬‬‬‬‬‬‬‬‬‬‬‬‬‬‬‬‬‬‬‬‬‬‬. Two experiments were designed to‬‬‬‬‬‬‬‬‬‬‬‬‬‬‬‬‬‬ ‬‬‬‬‬‬‬‬‬‬‬‬‬‬‬‬‬‬‬‬‬‬‬‬‬‬‬‬‬‬‬‬‬‬‬‬‬‬‬‬‬‬‬‬‬‬‬‬‬‬‬‬‬‬‬‬‬‬‬‬‬‬‬‬‬assess the impact of light quality on the plant. First, we investigated the physiological and photosynthetic response of *A*. *thaliana* to BL, AL, and RL lights compared it to FL by measuring leaf area growth, biomass content, Pn, and pigments content. Second, we tested whether changes in plant response to light quality is genotype specific by conducting the experiments across three *A*. *thaliana* accessions. Third, we investigated the potential induction of stress responses under AL by testing whether there are light quality-specific changes in the expression of marker genes involved in light-responsive photosynthetic process and enzymatic activity of antioxidants, as well as photosynthates content. Our findings expand the current understanding on physiological and photosynthetic responses of plants to light quality, in addition to identifying putative protective-mechanisms that may be induced to cope with lighting-stress in order to enhance plant stress tolerance.

## Materials and methods

### Plant materials and growth condition

Seeds of *A*. *thaliana* accessions Col-0, Est-1, and C24 were obtained from the Arabidopsis Biological Resource Center (ABRC; Columbus, OH, US). Seeds were placed in rockwool cubes (Grodan A/S, DK-2640, Hedehusene, Denmark) and incubated at 4°C for 2 days. White broad-spectrum light (FL; 4200 K, F72T8CW, Osram Sylvania, MA, US) were used as light sources for seed germination. Seedlings were hydroponically grown under FL for 21 days with the environmental condition of 24 h photoperiod, 23°C, 50% relative humidity, and ambient CO_2_ in a growth chamber (TC30, Conviron, Winnipeg, MB, Canada). Seed density was adjusted to limit treated plants from shadowing each other. FL was placed over the plant-growing surface area (49 cm × 95 cm) at a low photosynthetic photon flux density (PPFD) of 69 to 71 μmol·m^-2^·sec^-1^. PPFD was measured at the conjunction of a grid (square area 3 cm^2^) placed over the growing area. After 21 days, plants formed rosettes with nine (C24) and eleven (Col-0 and Est-1) leaves. To reach the same growth stage as Col-0 and Est-1 plants, C24 plants were allowed to grow for 23 days [37]. Fresh half-strength Hoagland nutrient solution [38] was provided every other day.

### Light treatment

After day 21 (Col-0 and Est-1) or 23 (C24), plants were transferred to their respective light treatment for 5 days, each with the same environmental conditions: 24 h photoperiod, 23°C, 50% relative humidity, and ambient CO_2_. 21-day old plants were randomly divided into four experimental groups and received treatments using light emitting diodes (LED) (VanqLED, Shenzhen, China) of BL (peak wavelength: 450 nm), AL (peak wavelength: 595 nm), and RL (peak wavelength: 650 nm). The fourth group was treated with FL (400–700 nm), as the control. The light spectra and PPFD were monitored daily by using a PS-300 spectroradiometer (Apogee, Logan, UT, US). PPFD was maintained at 69 to 71 μmol·m^-2^·sec^-1^ throughout the whole plant growth period. Fresh half-strength Hoagland nutrient solution [38] was provided every other day. Biological replicates were grown at different time points under the same environmental settings.

### Physical and biochemical analyses

#### Leaf area growth determination

Three plants per biological replicate were randomly selected for each measurement. Leaves from the selected plants were collected for the determination after treatment (5 days). Digital images of leaves were taken with a window size of 640 x 480 pixels and a camera-object distance of approximately 80 cm. The digital images were next used to determine leaf area growth using Image J software with default settings (Bethesda, MD, US), as described previously [39].

#### Biomass content determination

Three plants per biological replicate were randomly selected for each determination. Leaf samples from the selected plants were collected for the dry mass determination before (0 h) and after treatment (5 days). Leaves were dried at 80°C for 2 days until a constant mass was achieved (less than < 5% mass difference over a 2 h period).

#### Pigment content determination

Five plants per biological replicate were randomly selected for each assay. Leaf samples from the selected plants were collected for the determination after treatment (5 days). Methods and equations described by [40–42] were used to estimate the content of chlorophyll (Chl a and Chl b), carotenoids, and anthocyanin, respectively. Briefly, chlorophylls and carotenoids were extracted with 5 ml of 80% acetone at 4°C overnight, before centrifugation at 13,000 g for 5 min. Total anthocyanins were determined by extracting with 5 ml 80% methanol containing 1% HCl solvent at 4°C overnight, before centrifugation at 13,000 *g* for 5 min. The absorbance of the extraction solution was determined for Chl a (664 nm), Chl b (647 nm), carotenoids (440 nm), and anthocyanins (530 nm and 657 nm) using a UV–VIS spectrophotometer (UV-180, Shimadzu, Japan).

### Net photosynthetic rate determination

Net photosynthetic rate was monitored before (0 h) and after treatment (5 days) using the LI-6400XT Portable Photosynthesis System (LI-COR Biosciences, Lincoln, NE, US) equipped with a 6400–17 Whole Plant Arabidopsis Chamber (LI-COR Biosciences). To reduce potential measurement errors, three plants were grouped as a single sample for determinations [43]. To avoid mismatch between the light quality used by the LI-6400XT Portable Photosynthetic System, and the LED lights used for the treatments [44], measurements were taken inside the controlled-chamber, in which whole plants (still embedded in rockwool) were placed and illuminated with LEDs. As a precaution, parafilm was placed on top of the rockwool cube to maintain moisture within the root zone while measurements were recorded. The environmental conditions of the chamber were set as: 400 ppm CO_2_, 50% relative humidity, 23°C, and 400 μl min^-1^ flow rate. Each measurement was taken over 20 min, including 5 min in the dark and 10–15 min under a light treatment at 69–71 μmol·m^-2^·sec^-1^. A stable Pn reading was reached 10 min after illumination. Leaf area growth was determined to normalize Pn per unit leaf area growth. Measurements for three replicates (three plants per replicate, three replicates per treatment) were performed.

### Photosynthate content determination

Previous studies have reported that the diurnal cycle and developmental stage of plants, along with the stress response can affect the plant metabolism [45, 46]. Thus, we performed a time course assessment of 0, 1, 3, 5, and 7 days to determine the content of leaf photosynthates (proteins, starches, and lipids). Five plants per biological replicate were randomly selected for each measurement. Leaf samples from selected plants were collected for the determination prior (0 h) and after light treatments (1, 3, 5, and 7 days). Samples were immediately frozen in liquid nitrogen and stored at -80 ^∘^C, before they were used for determination. **Protein**: Total protein content was measured using the Pierce™ BCA Protein Assay Kit (Thermo Fisher Scientific, Rockford, IL, USA). As a standard, the absorbance of the bovine serum albumin was determined at UV/Vis: λ_max_ 562 nm. **Starch**: A previously described method [47] was used to estimate total starch content. **Lipids**: Previously described methods [48, 49] were used (with minor modifications) to estimate the total lipid content. Briefly, each sample was homogenized with (CHCl_3_/MeOH, 70:30 v/v), before centrifuged at 1000 rpm for 5 min. The collected supernatant was incubated for 30 min at 70°C in a boiling water bath. Next, (H_2_SO_4_: 1 ml) was added and heated for 20 min. Following 2 min cooling on ice, (H_3_PO_4_: 1.5 ml) was added and incubated for 10 min until a pink color developed.

### Antioxidative enzyme activity estimation

Five plants per biological replicate were randomly selected for each measurement. Leaf samples from selected plants were collected for the determination after treatment (5 days). Samples were immediately frozen in liquid nitrogen and stored at -80 ^∘^C, before they were used for determination. Methods described by [50, 51] were used to monitor the activity of SOD and APX antioxidative enzymes, respectively. Enzymatic activity was measured for 5 min at room temperature. The protein content in the supernatant was determined by the Pierce™ BCA Protein Assay Kit. The activity of SOD and APX was expressed as unit min^−1^ mg^−1^ protein.

### Gene expression analysis

#### cDNA synthesis

Changes in transcription of the interested genes were analyzed in *A*. *thaliana* Col-0 treated for 24 h under AL, RL, and FL. Leaf samples from selected plants were collected for the determination prior to treatment (0 h) and after treatment (2 h, 4 h, and 24 h). Samples were immediately frozen in liquid nitrogen and stored at -80 ^∘^C, before they were used for determination. Four biological replicates were examined. For each biological replicate, five *A*. *thaliana* plants were selected, and their leaves were pooled together to represent a biological replicate. Plants in each biological replicate were grown independently, and at different times. Total RNA was extracted from (100 mg) leaves using the Sigma Spectrum Plant Total RNA Kit (STRN50; Sigma, Seelze, Germany) according to the manufacturer’s protocol. A total of (2 μg) RNA per sample was treated with amplification grade DNase I (Invitrogen, Carlsbad, CA, USA) to remove any traces of genomic DNA contamination. RNA concentrations were measured before and after DNase I digestion with a NanoDrop ND-1000 UV-Vis spectrophotometer (NanoDrop Technologies, Wilmington, Delware, USA). The cDNA was synthesized using AffinityScript QPCR cDNA Synthesis Kit (Agilent, Tech., Santa Clara, USA).

#### Primer design

Primers for genes of interest (S1 Table) were designed using IDT software (https://www.idtdna.com/calc/analyzer) with the following criteria: Tm of 58–60°C and PCR amplicon lengths of 70 to 120 bp, yielding primer sequences 20 to 25 nucleotides in length with G-C contents of 40% to 50%. Specificity of the resulting primer pair sequences was examined using Arabidopsis transcript database with TAIR BLAST (http://www.arabidopsis.org/Blast/). Specificity of the primer amplicons was further confirmed by melting-curve analysis (30 amplification cycles by PCR and subsequent gel-electrophoretic analysis). Primer amplicons were resolved on (agarose gels, 2% w/v) run at 110 V in Tris-borate/EDTA buffer, along with a 1Kb^+^ DNA-standard ladder (Invitrogen, Carlsbad, CA, USA).

#### Quantitative real time-PCR (qRT-PCR) analysis

Real-time qRT-PCR was performed with a MX3000P qPCR System (Agilent, Tech., Santa Clara, CA, USA) using three biological and two technical replicates, as described previously [52]. Relative expression was conducted following the manufacturer’s recommendations with two reference genes gamma tonoplast intrinsic protein 2 (*TIP2*; AT3g26520) and actin 2 (*ACT2*; AT3g18780) and the Brilliant III SYBR Green QPCR master mix (Agilent, Tech., Santa Clara, CA, USA). Amplification was performed in a (20 μL) reaction mixture containing (160 nmol) for each primer, 1x Brilliant III SYBR Green QPCR master mix, (15μM) ROX reference dye, and (0.3 μL) of cDNA template. Amplification conditions were 95°C for 10 min (hot start), followed by 40 cycles at 94°C for 30 s, 60°C for 30 s, and 72°C for 30 s. Fluorescence readings were taken at 72°C, at the end of the elongation cycle.

#### Data analysis

Ct values were calculated with CFX-Manager and MX-3000P software. Relative expression changes (delta-delta Ct) were calculated according to [53] using *A*. *thaliana TIP2* (AT3g26520) and *ACT2* (AT3g18780) as reference genes. To avoid multiple testing, the p-values were only considered for 0 h with 24 h (a total of 12 genes and two light conditions). A gene was considered differentially expressed if *p <*0.05 and the fold change pattern at 24 h was consistent with those observed at 2 and 4 h.

### Statistical analysis

Differences between light treatments were tested using the two-tailed Student’s t-test. A two-way ANOVA was used to assess the effects of accession and different light treatments on leaf area growth, biomass content, Pn value, and pigments content. We observed similar patterns using the non-parametric tests of Wilcoxon-Mann-Whitney and Kruskal-Wallis tests (data not shown). ‬

## Results

### Effect of light quality and natural genotype variation on leaf area growth, biomass content, net photosynthetic rate, and pigment content in *A*. *thaliana*

**Fig 1:**
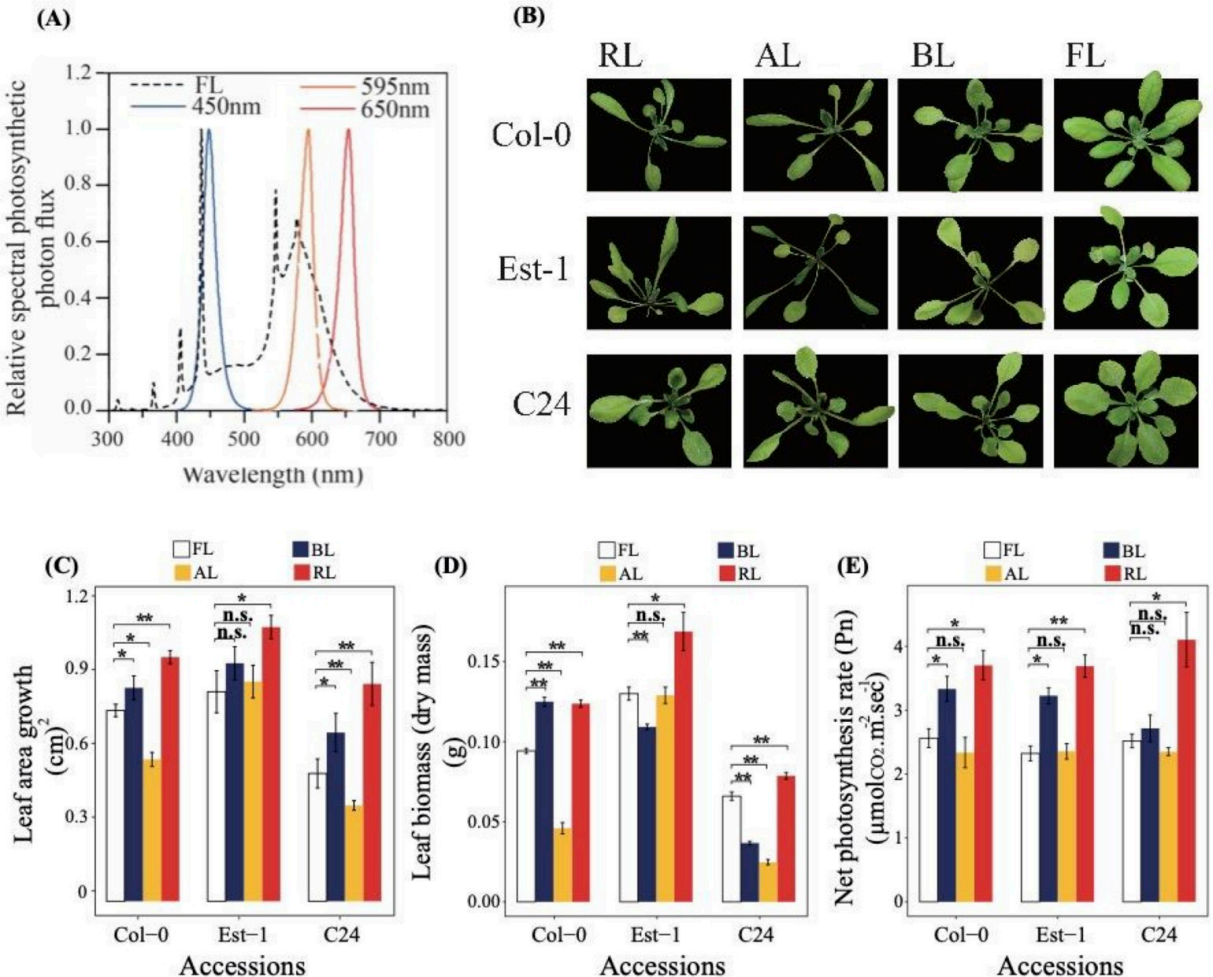
Effect of BL, AL, RL and FL on morphology, leaf area growth, biomass, and Pn of *A*. *thaliana* accessions. (A) Light emission spectra of LED light sources and FL. (B) Eleven-leaves stage *A*. *thaliana* accessions Col-0, Est-1, and C24 were grown hydroponically and treated for 5 days under narrow-spectrum BL, AL, and RL lights, as well as FL as control. (C) Leaf area growth. (D) Leaf biomass (dry mass). (E) Net photosynthetic rate (Pn) measured at 69–71 μmol m^-2^ sec^-1^. Data are expressed as mean values ± standard deviation (n = 3). Statistical analysis was performed against FL using a two-tailed Student’s t-test (n.s.: not statistically significant; *: *P <*0.05; **: *P <*0.01).

To assess the effect of light quality, 21-days-old plants (11 leave plants) of three *A*. *thaliana* accessions Col-0, Est-1, and C24 were randomly divided into groups and treated under narrow-spectrum light (BL, AL, and RL), along with FL as control (baseline), for 5 days at approximately 70 μmol m^-2^ sec^-1^ (Fig 1A and 1B). Summary of light quantity compositions emitted from FL and LEDs light sources are shown in Table 1. After 5 days of narrow-spectrum light treatments, leaf area growth, leaf biomass (dry mass), net photosynthetic rate, and pigment contents were measured across three *A*. *thaliana* accessions and compared with the baseline FL treatment (Fig 1C–1E and Table 2).

**Table 1:** Summary of light quantity compositions emitted from FL and LEDs light sources.

Wavelength range (nm)	PPFD percentage (%)			
	FL	BL	AL	RL
400–450	13.23	59.3	0	0
451–500	10.17	39.98	0	0
501–550	18.15	0.72	0	0
551–600	36.82	0	30.59	0.97
601–650	18.56	0	68.96	47.84
651–700	3.08	0	0.45	51.19

**Table 2:** Effect of BL, AL and RL on pigments content of *A*. *thaliana* accessions.

Accession	Parameters	BL	AL	RL	FL (control)
**Col-0**	Chl a	412.3 ± 42.9	314.6 ± 12.7	441.1 ± 27.2	381.9 ± 25.8
	Chl b	148.3 ± 13.0	125.0 ± 5.6	132.5 ± 7.9	147.6 ± 4.5
	Chl a: b	2.86	2.52*	3.17**	2.78
	Anthocyanins	77.8 ± 6.3	71.2 ± 7.7	72.3 ± 5.7	68.9 ± 5.7
** **	Carotenoids	97.8 ± 5.0*	84.7 ± 7.8	67.4 ± 8.0	76.1 ± 5.8
**Est-1**	Chl a	416.6 ± 34.1	325.7 ± 42.2	478.4 ± 11.2*	417.7± 16.9
	Chl b	142.6 ± 12.0	130.1 ± 15.9	149.7 ± 3.2	143.3 ± 10.6
	Chl a: b	2.95	2.51**	3.22*	2.91
	Anthocyanins	91.9 ± 1.5**	76.4 ± 3.2	75.5 ± 5.5	78.1 ± 0.6
** **	Carotenoids	95.5 ± 1.6*	91.7 ± 3.1	83.2 ± 4.3	80.1 ± 3.8
**C24**	Chl a	446.6 ± 21.7	359.7 ± 2.9	465.3 ± 9.1	405.4 ± 39.4
	Chl b	153.7 ± 9.3	145.7 ± 8.0	132.3 ± 9.8	129.9 ± 10.8
	Chl a: b	2.91	2.61**	3.22**	2.87
	Anthocyanins	89.7 ± 0.4**	76.5 ± 5.7	81.5 ± 4.2	79.2 ± 0.5
	Carotenoids	104.8 ± 8.3	93.0 ± 1.6	83.5± 0.5	87.0 ± 2.1

Data are expressed as mean values ± standard deviation (μg g^-1^ dry mass) (n = 5). Statistical analysis was performed against FL using a two-tailed Student’s t-test (*, *P <*0.05 and **, *P <*0.01).

**Fig 2:**
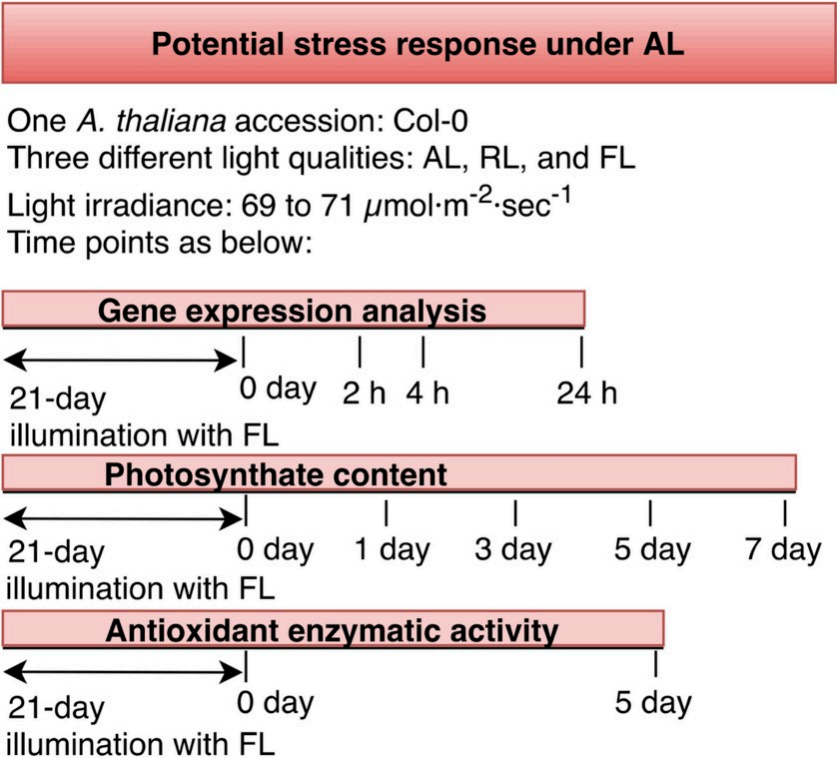
Flow diagram of study design to investigate the potential induction of stress response under AL including gene expression analysis, photosynthate content, and antioxidant enzymatic activity, in *A*. *thaliana* Col-0 under AL and RL.

Under RL, the leaf area growth was significantly increased across accessions (*P <*0.05; Fig 1C). Under BL, leaf area growth was significantly increased in C24 and Col-0 (*P <*0.05; Fig 2C), but the increase in the leaf area growth was not significant in Est-1. Under AL, leaf area growth showed a severe reduction in Col-0 and C24 (*P <*0.05; Fig 1C), while Al showed no change in Est-1. Petioles were noticeably elongated under AL (Fig 1B).

The leaf biomass significantly increased under RL across the three accessions (*P <*0.05; Fig 1D). Under BL, the leaf biomass was significantly decreased in Est-1 and C24 but increased in Col-0 (*P <*0.01; Fig 1D). Under AL, the leaf biomass was significantly lower in Col-0 and C24 (*P <*0.01), while it showed no change in Est-1 (Fig 1D).

As for the net photosynthetic rates (Pn), it significantly increased under RL across the accessions (*P <*0.05; Fig 1E). In contrast, there was no significant difference in Pn under AL (Fig 1E). Under BL, Pn significantly increased in Col-0 and Est-1 (*P <*0.05; Fig 1E) but remained unchanged in C24.

There was no significant difference in contents of chlorophyll a (Chl a) and chlorophyll b (Chl b) in Col-0 and C24 under the light quality of BL, AL, and RL (Table 2). In contrast, Chl a content significantly increased in Est-1 under RL (*P <*0.05; Table 2). Across accessions, Chl a: b content significantly increased, remained unchanged, and decreased under RL, BL, and AL, respectively (Table 2). Moreover, there was no significant difference in carotenoid and anthocyanin contents across the accessions under AL and RL. However, BL significantly stimulated carotenoids content in Est-1 and Col-0 (*P <*0.05; Table 2). Additionally, anthocyanins content significantly increased under BL in Est-1 and C24 (*P <*0.01; Table 2).

The two-way ANOVA analysis indicated significant effects of the light treatments for the determined parameters, except Chl b. Also, the interaction between light treatments and genotype was significant for leaf area growth and leaf biomass (*P <*0.01; Table 3).

**Table 3:** Summary of the two-way ANOVA analysis performed on *A*. *thaliana* accessions and effects on the determined parameters.

Parameter	Light (L)	Genotype (G)	LxG Interaction
Leaf area growth	1.60 x 10^−12^ ***	3.27 x 10^−14^ ***	0.002279 **
Leaf biomass (dry mass)	< 2.20 x 10^−16^ ***	7.81 x 10^−11^ ***	2.11 x 10^−7^ ***
Net photosynthetic rate (Pn)	5.05 x 10^−10^ ***	0.4096	0.2586
Chlorophyll a	0.0002218 ***	0.3171671	0.9187594
Chlorophyll b	0.1194	0.5281	0.2175
Anthocyanins	0.01634*	0.02138*	0.91437
Carotenoids	0.0001152***	0.0232754*	0.7358857

Shown are p-values for each set of tests.

*, Significant effects (*P <0.05

** *P <*0.01

*** *P <*0.001).

### Changes in transcription of photosynthetic marker genes, content of photosynthates, and activity of antioxidant in *A*. *thaliana* Col-0 under AL and RL

The severe reduction in leaf area growth and biomass, along with unchanged levels of Pn in Col-0 and C24 under AL suggested that amber light has mismatched effects on photosynthetic activity and photomorphology. Further to this, although chlorophyll contents under AL were 10–20% lower than the FL, both light treatments triggered similar photosynthetic activity, which implies that amber light has unidentified mechanisms in the photosynthetic process. To identify the mechanisms that amber light triggers within plants, we next explored transcriptional changes in marker genes associated with the photosynthetic light reaction and photo-protective mechanisms, photosynthates content and antioxidant enzymatic activity in Col-0 under AL (Fig 2). Among three accession, Accession Col-0 was chosen for the transcription analysis, as it is the most common *A*. *thaliana* accession in conducting biological analysis. In addition to AL and FL (as control), changes were investigated under RL, as RL-treated plants showed opposing changes in leaf physiological phenotypes compared to AL.

**Fig 3:**
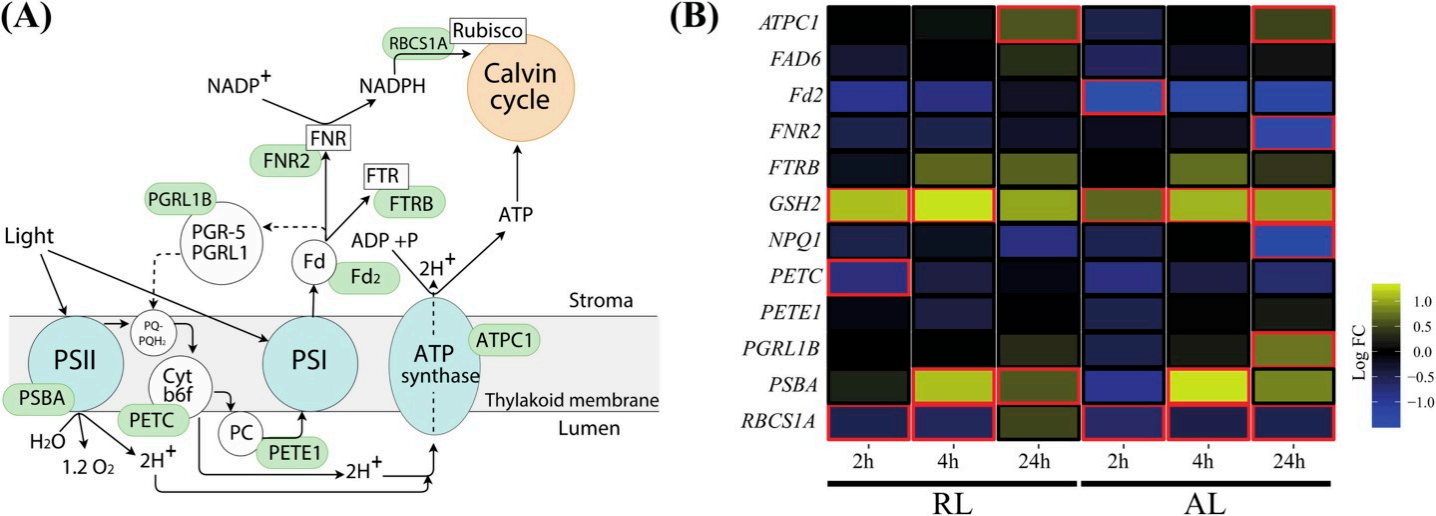
A schematic model of light-responsive photosynthetic process and effect of AL and RL on transcription of selected genes in *A*. *thaliana* Col-0. (A) Genes of interest are highlighted in green. (B) Transcription of genes implicated in the light-responsive photosynthetic process that is located within the thylakoid membrane. A time course assessment prior to treatment (0 h), and after treatment (2, 4, and 24 h) of AL and RL was performed, compared to FL. Four biological replicates were examined. For each biological replicate, five *A*. *thaliana* plants were selected, and their leaves were pooled together to represent a biological replicate. Plants in each biological replicate were grown independently, and at different time.

Gene expression analysis indicated a significant increase in transcription level of ATP synthase gamma chain 1 (*ATPC1*;member of ATP synthase complex) and proton gradient regulation Like 1 (*PGRL1B*;member of CET complex), after 24 h treatment under AL (*P <*0.05; Fig 3B). *ATPC1* transcription significantly increased after 24 h treatment under RL (*P <*0.05; Fig 3B). No significant difference, after 24 h treatment, was observed in the transcription level of the selected marker genes associated with linear photosynthetic electron transfer (i.e., ferredoxin-2 (*Fd2)*, plastocyanin *(PETE1)*, and cytochrome b6f complex (*PETC*) under both AL and RL (Fig 3B). After the 24 h treatment, transcription of ferredoxin-NADP+-oxidoreductase *(FNR2)* was significantly decreased under AL (*P <*0.05; Fig 3B), while it remained unchanged under RL (Fig 3B). The transcription level of ribulose bisphosphate carboxylase small chain *(RBCS1A)* was significantly reduced at 2 h and 4 h treatment under both AL and RL (*P <*0.05; Fig 3B). The *RBCS1A* transcription level significantly was downregulated under AL (*P <*0.05; Fig 3B). However, *RBCS1A* transcription level recovered after the 24 h treatment under RL (Fig 3B).

All data were normalized to the housekeeping genes; gamma tonoplast intrinsic protein 2 (*TIP2*; AT3g26520) and actin 2 (*ACT2*; AT3g18780). Red borders represent significant changes in expression (*P <*0.05). Studied genes include: ATP synthase gamma chain 1, *ATPC1* (AT4g04640); fatty acid desaturase 6, *FAD6* (AT4g30950); ferredoxin-2, *Fd2* (AT1g60950); ferredoxin-NADP+-oxidoreductase, *FNR2* (AT1g20020); (Fdx)-thioredoxin (Trx)-reductase, *FTRB* (AT2g04700); glutathione synthetase, *GSH2* (AT5g27380); PSII nonphotochemical quenching, *NPQ1* (AT1g08550); cytochrome b6f complex (Cyt b6f), *PETC* (AT4g03280); plastocyanin, *PETE1* (AT1g76100); proton gradient regulation Like 1, *PGRL1B* (AT4g11960); photosystem II protein D1, *PSBA* (ATCG00020) and ribulose bisphosphate carboxylase small chain, *RBCS1A* (AT1g67090).

To confirm changes in the ATP synthase and CET complex under AL, we leveraged available proteomics data where eleven-leaves plants of *A*. *thaliana* Col-0 were grown under AL and RL for 5 days. Consistent with the observed transcriptomic data, a significant increase in the level of protein abundance was observed for both CET complex (*P <*1.3 x 10^−12^; S1A Fig) and ATP synthase (*P <*2 x 10^−4^; S1B Fig) under AL compared to RL.

### Regulation patterns of *PSBA*, *NPQ1*, *GSH2* and *FAD6* transcripts in *A*. *thaliana* Col-0 under AL and RL

The transcription level of photosystem II protein D1 (*PSBA)* was significantly upregulated at 4 h and 24 h treatment under RL (*P <*0.05; Fig 3B). Under AL, the transcription level of *PBSA* showed a similar increase after the 4 h treatment (*P <*0.05; Fig 3B); However, its transcription level was reduced to a comparable level with FL after the 24 h treatment under AL. After the 24 h treatment, the transcription level of PSII nonphotochemical quenching *(NPQ1)* was significantly downregulated under AL (*P <*0.05; Fig 3B), while it remained steady under RL. Between the 2 h and 4 h treatment, the transcription level of *GSH2* gradually increased under both AL and RL (*P <*0.05; Fig 3B) but reduced to a comparable level with FL after the 24 h treatment under RL. No significant difference was observed in the transcription level of fatty acid desaturase 6 (*FAD6)* after the 24 h treatment under either AL or RL (Fig 3B).

### Photosynthates content in *A*. *thaliana* Col-0 under AL and RL

**Fig 4:**
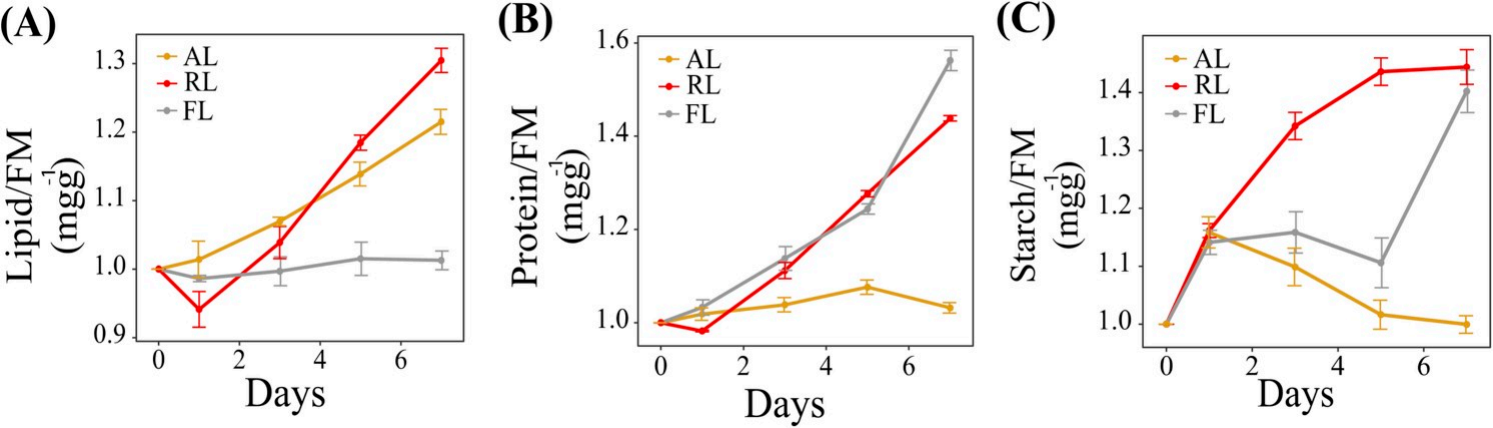
Effect of AL and RL on photosynthates content in *A*. *thaliana* Col-0. (A) Lipid; (B) Protein; (C) Starch. Data are expressed as mean values ± standard deviation (n = 5). Statistical analysis was performed against FL using a two-tailed Student’s t-test.

Photosynthates accumulation was probed in Col-0 treated under AL, RL, and FL. Total lipid, protein and starch were measured at days 0, 1, 3, 5, and 7 (Fig 4). The lipid content gradually increased under AL and RL (*P <*0.05; Fig 4A). The content level of proteins and starches increased under RL but decreased under AL (*P <*0.05; Fig 4B and 4C).

### Antioxidative enzyme activity in *A*. *thaliana* Col-0 under AL and RL

**Fig 5:**
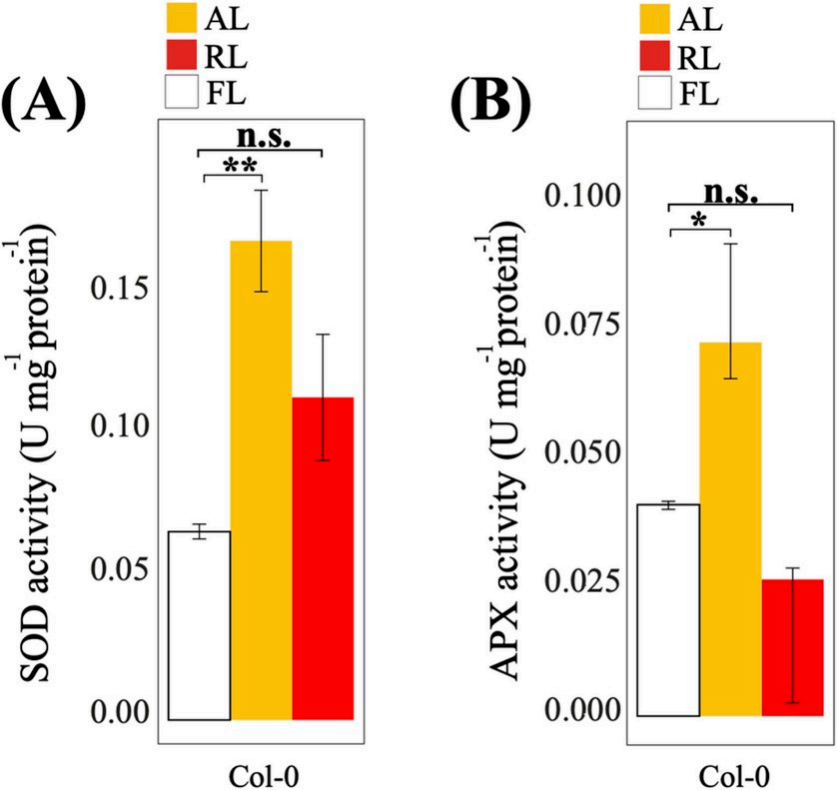
Effect of AL and RL on SOD and APX activity in *A*. *thaliana* Col-0. (A) Superoxide dismutase (SOD) activity. One unit of SOD activity was defined as the amount of enzyme required to result in a 50% inhibition of the rate of reduction at 550 nm in 1 min. (B) Ascorbate peroxidase (APX) activity. One unit of APX activity was defined as the amount of enzyme required to oxidize 1 μmol of ascorbate at 290 nm in 1 min. Enzymatic activity was measured for 5 min at room temperature and data are expressed as mean values ± standard deviation (n = 5). Statistical analysis was performed against FL using a two-tailed Student’s t-test (n.s., not statistically significant; *, *P <*0.05; **, *P <*0.01).

We examined the antioxidative activity of superoxide dismutase (SOD) and ascorbate peroxidase (APX) enzymes in Col-0 treated under AL, RL, and FL (Fig 5). After the 24 h treatments, activity of both antioxidants was significantly increased under AL (*P <*0.05; Fig 5), while no significant changes were observed for either of these enzymes when plants were treated under RL.

## Discussion

In this work, we investigated the impact of light quality BL, AL, and RL on leaf growth and photosynthetic response across three *A*. *thaliana* accessions Col-0, Est-1, and C24. The analyses clearly demonstrate the significant impact of light quality on leaf area growth, biomass content, and pigments accumulation (chlorophylls, carotenoid, and anthocyanin). The results indicate that light quality significantly influences Pn across accessions, consistent with the reported results that leaf photosynthetic reaction is wavelength-dependent in higher plants [54].

### Importance of geographic habitats on light quality response of leaf growth and biomass

The selected accessions Col-0, Est-1, and C24 have different geographic habitats; C24 originated from a part of Europe (Portugal), Est-1 from Northern Asia (Russia), and Col-0 from United States (Columbia). Therefore, we took into account differences in geographical range for these accessions resulted in a high degree of divergence in photosynthetic characteristics to light [35]. The most extreme responder in leaf area growth and leaf biomass analyses was Est-1 from Russia. It is worth pointing out that the two Col-0 and C24 accessions highlighted here as weak responders, they elongated very quickly under AL and thus may not be true candidates for weak responders. Previous studies have found negative correlations between hypocotyl height and latitude of accession origin in European *Arabidopsis* accessions [55, 56], suggesting that this natural variation in light sensitivity could be a result of adaptation to the north-south gradient in ambient light intensity.

The results of the study described here emphasize the strength of explicitly incorporating LxG interactions into the leaf area growth and leaf biomass content across the accessions. Importantly, as further elaborated below, the genotype-specific responses in leaf area growth and biomass content were observed exclusively under AL and BL, while the three accessions exhibited similar patterns of changes under RL. Our findings are consistent with previous reports on different accessions and light quality treatments, and underscores the importance of considering the natural habitat effect in characterizing the impact of light quality on leaves [57].

Leaf development varied between accessions such that the overall dynamic of growth and biomass were different. For example, we took efforts in synchronizing leaf growth stage in the accessions, resulting in the C24 plants being grow for 23 days to reach the same leaf stages of the plant. Some of the observed variation in leaf growth response could be simply a manifestation of the different time-course between accessions. These differences between accession can be significant and have the potential to enhance our understanding of the ecological role of specific adaptations.

### Findings on BL supports its role on activation of protective pigments

BL induced higher leaf area growth across three accessions. However, its impact on biomass production is accession-dependent, and may be caused by accessory pigment accumulation (anthocyanins). Under BL, only Col-0 showed an increase in biomass, as opposed to Est-1 and C24, which showed a decrease in biomass. It was observed that BL induced a significantly higher concentration of anthocyanins in Est-1 and C24 than Col-0. These results imply that the impact of wavelength on accessory pigment accumulation is accession-dependent, and that this difference in accessory pigment accumulation consequently leads to differences in biomass production across accessions. Anthocyanin is a photo-protective pigment, which protects plant and its chloroplast membrane by absorbing blue light and against photo-oxidation [58, 59]. Higher concentration of anthocyanin accumulating in a plant results in lower BL interception, which consequently lead to lower biomass production over the long term. Further to this, in this study, we found the ratio of Chl a:b is similar under BL and FL across accessions. This consistency in Chl a:b, suggests a lack of photosystems reconfiguration under BL [60, 61]. Our results confirm the role of BL in stimulating anthocyanin content in plants and protecting them from light stress [62]. Plants activate photo-protective mechanisms under BL to cope with a potential induced-light stress, resulting in an increased accumulation of photo-protective pigments [58, 59]. Notably, we found different patterns in content of anthocyanin accumulation in the accessions. Results showed that anthocyanin accumulation can be triggered at low BL (~70 μmol m^-2^ sec^-1^), which suggests that this protective mechanism against BL can vary based on the accession (i.e. natural adaptations) and can be triggered under low light. Further investigation on these two accessions on BL with a wide range of BL intensity is required. Our results thus encourage future studies analyzing this trait using BL with a wide range of BL intensity to further advance our understanding of the underlying mechanisms.

### Plants showed high antioxidative and photo-protective under AL

AL had no impact on the photosynthetic activity across the three accessions compared to FL; yet it induced the poorest morphological traits. Col-0 and C24 showed a severe reduction ‬‬in leaf area growth and biomass, while Est-1 was unaffected. These two accessions (Col-0 and C24) showed a clear elongation of petioles under AL, which suggests that leaf resources are redirected from leaves to petioles as insufficient lighting conditions under AL were performed in this study [63]. However, the results on transcriptional changes and photosynthates content showed the opposite responses to the morphological traits.

The photosynthates, including proteins and starches, showed lower content in leaves of plants treated under AL. A downregulation of *RBCS1A* (small subunit of Rubisco) transcription was also observed in the leaves treated under AL. A lower accumulation of proteins was previously observed under AL [64], suggesting a positive contribution of downregulated Rubisco genes, as it is the main protein in leaves. A lower content of carbohydrates under stress conditions has been observed before in *A*. *thaliana* [65]. Future work is needed to explore if a reduced conversion of light energy into chemical energy has occurred in the photosynthesis process under AL.

High capacity for lipids accumulation was observed for plants treated under AL. Lipid accumulation had been previously linked to oxidative stress [66]. suggesting an increase in lipophilic antioxidants content such as tocopherols, which play an important role in the scavenging of singlet oxygen [67]. Moreover, we found a significant increase in both expression and enzymatic activity of antioxidants under AL. Plants stimulate antioxidative mechanisms to protect the photosynthetic apparatus from incurring damage via ROS detoxification [30, 68]. Our results on photosynthates thus suggest that plants tried to cope with a potential ROS stress condition under AL.

A significant upregulation in glutathione biosynthesis, transcription level of *PGRL1B*, *ATPC1*, and marker genes associated with ATP synthase and CET complex was observed. In agreement with this result, a significant increase in the expression of ATPC1 at the protein level was recently reported in *A*. *thaliana* Col-0 treated with 595 nm light [69]. CET plays an important role to protect plants under high light and other stress environments [70]. During CET, electrons are cycled around PSI and protons are translocated to generate a proton gradient across the thylakoid membranes [71]. In addition to contributing ATP synthesis, another function of a generated proton gradient is to dissipate excess energy as heat from the PSII antennae [72]. Further to this, an upregulation of CET and ATP synthase suggests of an accelerated rate of PSII repair through elevated ATP synthesis [73, 74]. As such, the results on photosynthates and at the transcription level under AL both suggest that AL, even at low light, induces protective mechanisms of photosystems related to light stress, which consequently triggers low protein and starch accumulation and result in poor morphological traits.

One possible hypothesis for the conflicting AL responses can be explained by the detour effect [75, 76], where a major part of AL transmitted into the leaf is reflected within leaf tissues and re-absorbed by unsaturated chlorophylls multiple times, which leads to an observed light stress response. Due to the nature of the high absorbing efficiency of the chloroplast, nearly 90% of BL and RL are absorbed at the leaf surface and their detour effect is small [76, 77]. While for the wavelength within 500–600 nm [i.e. green light (GL) and AL] that are less absorbed by chloroplast, its light path can increase by several folds and this results in its increased/overexpressed photosynthetic activity through light absorption by unsaturated chloroplast. Although there is no study reporting the underlying mechanisms triggered by AL, several studies have observed the impact of supplemented GL and AL on photosynthetic activity and plant productivity in horticultural plants, which reinforces our hypothesis on the increased photosynthetic activity under AL. Further to this, the aggressive suppression responses on morphological traits in *A*. *thaliana* under AL, opposed to the positive impact on plant development, is expected as *A*. *thaliana* is a low light plant. They are more sensitive to the change in light properties. Overall, our results suggest AL as a potential light source in activating the potential of increased plant productivity efficiently, but it requires high control on its intensity. This study clarifies why AL alone induces overexpressed high photosynthetic activity yet results in poor plant development.

### RL modulated plant adaptation and energy assimilation

The leaf area growth was significantly increased under RL across all accessions, which in turn enabled a greater light interception by the leaves [78]. This agrees with the increased Pn that was observed across accessions. These observations along with a significant increase of leaf biomass suggests proper plant adaptation under RL across accessions. We found a significant increase in the Chl a: b under RL across accession. Chl a is mainly concentrated around PSI and PSII, whereas Chl b is most abundant in light-harvesting complexes [79]. An increase in Chl a: b can increase the likelihood of an efficient electron transfer system within the chloroplast membrane [80]. This, in turn, could positively influence the photosynthetic performance in plants under RL. Considering that timely synthesis of D1 protein is key to maintain the PSII function and consequently, photosynthetic performance in leaves [25]. An increasing trend of *PSBA* expression was observed in plants under RL. The *PSBA* gene is critical for the *de novo* synthesis of the D1 protein during PSII repairs [81, 82]. Therefore, upregulated transcription of *PSBA* gene could play an important role in accelerating the process of D1 protein turnover under RL. Plants showed that leaf photosynthates (starches, lipids, proteins) increased under RL. Overall, our results present RL as an efficient light source in helping the leaf energy assimilation process, resulting in an increased leaf growth, photosynthetic performance, and photosynthates content in plants.

## Supporting information

S1 File (ZIP)

S1 Table List of primers sequences used in qPCR experiments. (DOCX)

S1 Fig Proteins involved in ATP synthase and CET complex of *A*. *thaliana* Col-0 are upregulated under AL (595 nm) compared to RL (650 nm). In this experiment, eleven-leaves plants were grown under AL and RL for 5 days (three biological replicates per light condition). A) The expression pattern of protein members involved in Cyclic electron transfer (CET) complex. B) The expression pattern of protein members involved in ATP synthase complex. Expression levels for each protein is normalized to have mean of zero and standard deviation of one. Yellow or blue color indicates upregulation or downregulation, respectively.
(DOCX)
