# Supplementary material for: The effect of light quality on plant physiology, photosynthetic, and stress response in Arabidopsis thaliana leaves
Source: PLoS One. 2021 Mar 4;16(3):e0247380. doi: 10.1371/journal.pone.0247380 (PMC7932170; doi:10.1371/journal.pone.0247380)
Supplement: S1 Table — (DOCX) [file pone.0247380.s002.docx]

**S1 Table. List of primers sequences used in qPCR experiments**

| Gene Name | Forward Primer | Reverse Primer | Accession number |
| --- | --- | --- | --- |
|  | Sequence (5'-3') | Sequence (5'-3') |  |
| *FNR2* | TGTGTGGACTCAAGGGAATGGA | CTCTGCCTTCTTCAACTGCTTCTTG | AT1G20020 |
| *PETE1* | ACCGTCACCATCCCTTCTTTCA | ACTGCGATGACACCGAAATCCTT | AT1G76100 |
| *PETC* | GTATTCCAGCAGACAGAGTTCCAGA | AGGGACAAGCATGTAGCCAGTA | AT4G03280 |
| *PGRL1B* | AGTGTCCTGCTCCCTTTACCCAT | TGCTCTCAACTTCTTCCCCACCCA | AT4G11960 |
| *ATPC1* | AGTAGCTCTCGTTGTCGTCACC | TCTTGCCCACGCTAATGACTGT | AT4G04640 |
| *Fd2* | TTCATTCATCCGTCGTTCCCCA | ACGAGCGGTGCCTGATTTGA | AT1G60950 |
| *PSBA* | AGTTTCCGTCTGGGTATGCG | TAAAAAGGGAGCCGCCGAAT | ATCG00020 |
| *RBCS1A* | TCGGATTCTCAACTGTCTGATG | ATTTGTAGCCGCATTGTCCT | AT1G67090 |
| *FTRB* | CTCGATGAATCTTCAAGCTGTTTC | CAAAGCGGTGCACCATATGAATC | AT2G04700 |
| *FAD6* | ACTCTCGCCTTCCTACCACTTG | CTCAAACTCCTCTGGCGGAACT | AT4G30950 |
| *NPQ1* | TGGGAGATCCTCACGTCCTTT | CGAGTTTTAGCAACAGCGGAGC | AT1G08550 |
| *GSH2* | TTGCTACCAACTGCATTCCCAGA | TGCCATCCAAGCTAACACGATCA | AT5G27380 |
| *TIP2* | CGCCGCTTGTTTCCTCCTTA | AAGACGAGAGCGTTTAATGATCCGA | AT3G26520 |
| *ACT2* | CTTGCACCAAGCAGCATGAA | CCGATCCAGACACTGTACTTCCTT | AT3G18780 |
