# Supplementary material for: The effect of light quality on plant physiology, photosynthetic, and stress response in Arabidopsis thaliana leaves
Source: PLoS One. 2021 Mar 4;16(3):e0247380. doi: 10.1371/journal.pone.0247380 (PMC7932170; doi:10.1371/journal.pone.0247380)
Supplement: S1 Fig — In this experiment, eleven-leaves plants were grown under AL and RL for 5 days (three biological replicates per light condition). A) The expression pattern of protein members involved in Cyclic electron transfer (CET) complex. B) The expression pattern of protein members involved in ATP synthase complex. Expression levels for each protein is normalized to have mean of zero and standard deviation of one. Yellow or blue color indicates upregulation or downregulation, respectively. (DOCX) [file pone.0247380.s003.docx]

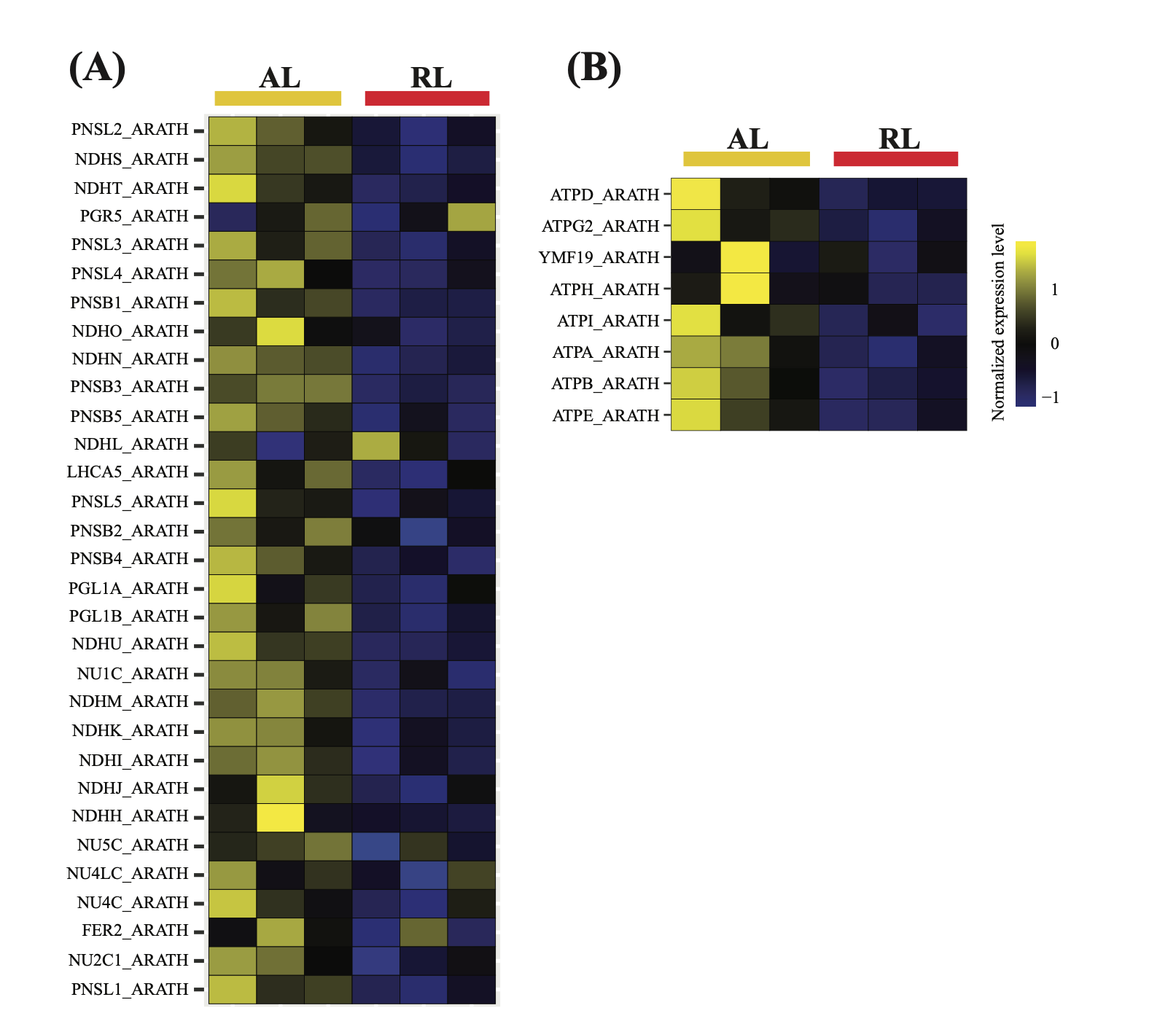


**S1 Fig. Proteins involved in ATP synthase and CET complex of *A. thaliana* Col-0 are upregulated under AL (595 nm) compared to RL (650 nm).**

In this experiment, eleven-leaves plants were grown under AL and RL for 5 days (three biological replicates per light condition). (A) The expression pattern of protein members involved in Cyclic electron transfer (CET) complex. (B) The expression pattern of protein members involved in ATP synthase complex. Expression levels for each protein is normalized to have mean of zero and standard deviation of one. Yellow or blue color indicates upregulation or downregulation, respectively.
